# The immune hunger games: the effects of fasting on monocytes

**DOI:** 10.1038/s41423-023-01033-w

**Published:** 2023-05-10

**Authors:** Jorge Domínguez-Andrés, Holger Reinecke, Yahya Sohrabi

**Affiliations:** 1 Department of Internal Medicine and Radboud Center for Infectious Diseases, Radboud University Nijmegen Medical Centre, 6500HB Nijmegen, the Netherlands; 2 Department of Cardiology I-Coronary and Peripheral Vascular Disease, Heart Failure, University Hospital Münster, Westfälische Wilhelms-Universität, Münster, Germany; 3 Institutes of Molecular Genetics of the Czech Academy of Sciences, Prague, Czech Republic; 4 Department of Medical Genetics, Third Faculty of Medicine, Charles University, Prague, Czech Republic

**Keywords:** Monocytes and macrophages, Innate immunity

Currently, different types of fasting are becoming increasingly popular for their potential health benefits, particularly in improving cardiometabolic diseases. However, how these practices affect immune function is not well understood. In a recent study published in *Immunity*, Janssen et al. delve into the complex relationship among fasting, refeeding, and the immune system. While fasting caused monocyte homing in bone marrow (BM), refeeding escalated monocyte counts in the circulation but altered immune responses to bacterial infection [1].

Our bodies have a remarkable ability to limit energy expenditure during nutrient scarcity, particularly among certain types of immune cells, such as monocytes. Monocytes are energetically costly due to their short half-life, massive daily production in the bone marrow, and reliance on myelopoiesis for replenishment. In their study, the authors demonstrated that during fasting, monocytes migrate back to the BM, where they are thought to hibernate, extending their lifespan and conserving energy [1].

**Fig. 1:**
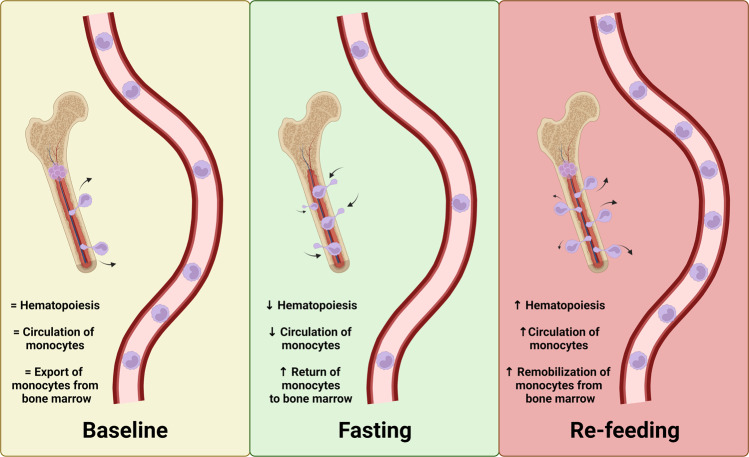
Monocyte dynamics during fasting and refeeding. The figure illustrates the key stages of monocyte behavior in response to fasting and refeeding. In the baseline condition (yellow), monocytes circulate in the bloodstream, and hematopoiesis occurs in the bone marrow. During fasting (green), monocytes migrate back to the bone marrow (BM). Increased number of cells in MB deacreases hematopoiesis. Upon refeeding (red), monocytes are remobilized from the bone marrow into the circulation and hematopoiesis resumes. Arrows indicate the direction of events across the different stages. The figure was created with BioRender.com

This phenomenon raises several questions, such as how monocytes survive in the BM during fasting and whether a mechanistic link exists between the remobilization of circulating monocytes and the reduction of hematopoiesis during fasting. Intrestingly, the central nervous system (CNS) plays a role in orchestrating large-scale leukocyte shifts. The authors propose a sequence of events occurring during fasting and refeeding that link the hypothalamic‒pituitary‒adrenal (HPA) axis with monocytes and BM [1]. Extended fasting induces a stress response mediated by corticosteroid (CORT) via the HPA axis. Fasting leads to increased levels of CORT that binds to the monocytic glucocorticoid receptor NR3C1, which in turn increases CXCR4 expression on monocytes, promoting their migration to the BM. Upon refeeding, CORT levels are normalized, and monocytes return to the circulation (Fig. 1). However, returning monocytes are transcriptionally distinct and chronologically older, which alter their function and ability to respond to an infection [1]. The effect of corticotropin-releasing hormone and neuron-mediated leukocyte shifts on immune responses against autoimmunity and viral infection have been shown previously [2]. Increasing evidence shows that different kinds of stresses induce the activation of the sympathetic nervous system, which modulates hematopoiesis and leukocyte hemostasis [3, 4]; however, the mechanistic foundation of how neuronal signals precipitate fasting-induced monocyte hemostasis is not clear. In addition, it was shown that the activation of hepatocyte-derived low-energy sensing AMPK is responsible for shutting down monocyte mobilization during fasting in a CCL2/PPARa-dependent manner [5].

These findings may have significant implications for a broad audience, including those who practice fasting for health benefits, as well as medical professionals and researchers. However, it is essential to note that the research focuses on prolonged fasting and refeeding, which may be more relevant to situations of severe food scarcity, malnutrition, or eating disorders, rather than intermittent fasting practices, which are commonly adopted by individuals seeking health benefits [6]. Moreover, considering the increasing number of people in the world suffering from hunger, these data stress the destructive effect of hunger and malnutrition on the immune system. Nevertheless, understanding the impact of fasting on immune function is crucial for both the general public and the scientific community.

There may be potential benefits to reduce the rate of hematopoiesis through practices such as prolonged fasting, exercise, better sleep hygiene, or improved diet [1]. Beyond such homeostatic control, decreasing health, poor sleep quality, high pain scores, hunger, and less physical activity enhance stress, which in turn activates the sympathetic nervous system and alters cell hemostasis [3]. This suggests that improving mental health conditions, psychological interventions, or pharmacological approaches to manage stress may have an impact on decreasing the risk of alteration in the host immune response. In addition, recent data indicate that accelerating the rate of leukocyte production may precipitate clonal hematopoiesis, reducing hematopoietic diversity and conferring a heightened risk of cardiovascular disease [4, 7]. Measures aimed at reducing hematopoiesis could provide long-term benefits by preserving a diverse, nonclonal hematopoietic pool. Future research should also consider the potential relationship between fasting and two other factors that play a role in immune cell hemostasis: stress and circadian rhythms [2, 3].

The study by Janssen et al. also raises interesting questions about the potential consequences of fasting on infection and disease outcomes. While several studies have suggested that short-term fasting can boost immune function and protect against certain infections, the effects of prolonged fasting and refeeding on the immune system’s ability to respond to pathogens remain unclear. Increasing evidence has shown that fasting can have broader but distinct effects on the immune system and other leukocytes function such as T cells amd B cells, other essential component of the immune response. Although calorie restriction triggers memory T-cell homing to the bone marrow and promotes survival and protective function [8], repeated fasting has deleterious effects on the levels of B cells in Peyer’s patches, and their survival is drastically reduced, which attenuates antigen-specific IgA responses [9]. Furthermore, the type and duration of fasting may lead to different outcomes. For instance, intermittent fasting improves chronic inflammatory diseases such as atherosclerosis [6], and time-restricted feeding has a beneficial impact on NAFLD but a deleterious impact on early atherosclerosis [10]. These effects may be mediated through similar mechanisms involving the HPA axis and CORT levels, suggesting a coordinated response to nutrient scarcity that influences multiple aspects of immune function. Moreover, CNS activation triggers myelopoiesis, which may accelerate the number of circulating monocytes following refeeding [4]. Altogether, the data indicate complex and possibly cell- and tissue-specific responses according to fasting conditions.

The changes in monocyte distribution and function observed in this study could have significant implications for the immune system’s capacity to mount an effective response during periods of fasting and refeeding. It is also evident that different forms and lengths of fasting may cause distinct and opposite effects on immune cells and various organs. Further research is warranted to determine the specific effects of these practices on susceptibility to infections and other immune-related conditions.

Additionally, the current data offer a valuable perspective on the potential impact of fasting on inflammatory diseases. Understanding the effects of fasting on immune cell behavior, particularly in the context of inflammation, could have significant implications for the management of these conditions. The returning monocytes observed after refeeding are transcriptionally distinct and chronologically older, which could affect their ability to respond to inflammatory stimuli. This raises the question of whether fasting and refeeding might exacerbate or ameliorate the symptoms of inflammatory disorders, such as cardiometabolic diseases, rheumatoid arthritis, inflammatory bowel disease, and asthma.

The current paper also highlights the individual variability in response to fasting and refeeding. In addition, factors such as age, sex, genetic background, and overall health status may influence the reaction of the immune system to these practices, leading to distinct outcomes in different individuals. As personalized medicine becomes increasingly prominent, understanding the individual factors that affect the response to fasting and refeeding could help optimize these practices for specific populations, maximizing their health benefits while minimizing potential risks.

Furthermore, the authors underscore the need for additional studies investigating the long-term effects of fasting and refeeding on immune function. While the current study provides valuable insights into the immediate consequences of these practices on monocyte distribution and function, the long-term implications remain less clear. Future research should explore the durability of these effects and whether repeated cycles of fasting and refeeding might lead to lasting changes in immune function, either beneficial or detrimental. Furthermore, metabolic shifts cause a long-term effect on innate immune cells; it needs to be clarified whether the energy-saving state during fasting has any persistent consequences on cell function. It is also crucial to determine the healthiest type of fasting regarding the duration and intervals.

In conclusion, the study by Janssen et al. sheds light on the complex relationships between fasting and the immune system. While the research has limitations in its direct translation to human physiology, it provides valuable insights into the potential costs of refeeding following prolonged fasting and how fasting and refeeding affect monocyte immune function and distribution. Further research is needed to explore the mechanisms underlying these findings and their implications for human health, dietary recommendations, and potential therapeutic interventions. This study and earlier studies may build an initial foundation to develop a combined immunomodulating approach through diet control and pharmacological means to control particular diseases. The data also mark an essential step toward a more comprehensive understanding of the “fast and furious” nature of monocyte behavior and its role in maintaining immune function during periods of fasting and refeeding.
